# The Castration Bill

**Published:** 1909-03

**Authors:** 


### Selected.

### The Castration Bill.

A bill to be entitled "An Act to amend Article 639 of Chapter 7, Title XV of the Penal Code of the State of Texas, in reference to the punishment for rape, and providing who shall perform castration, and fixing a fee therefor, and declaring an emergency."

*Be it enacted by the Legislature of the State of Texas:*

SECTION 1. That Article 639 of Chapter 7, Title XV of the Penal Code of the State of Texas, be and the same is hereby amended so as to read as follows:

Article 639. Who ever shall be guilty of rape shall be punished by death or by confinement in the penitentiary for life, or for any term of years not less than two, or by castration, or by such imprisonment and castration in the discretion of the jury; provided that punishment by castration shall apply only in case of rape by force, threats or fraud.

SEC. 2. Castration as provided for in the preceding section shall be performed by the county physician of the county where such conviction is had, if there be such physician; if there be no such physician such castration shall be performed by a competent physician to be appointed by the court trying such case. The physician performing the castration as provided for in this act shall receive therefor the sum of twenty-five dollars, to be paid by the State upon the certificate of the judge trying said cause.

SEC. 3. The fact that the crime of rape is rapidly increasing and that the additional penalty provided for in this act will in a large measure deter the rape fiend, creates an emergency and imperative public necessity, requiring that the constitutional rule providing that bills be read on three several days in each house be suspended, and that this act take effect and be in force from and after its passage, and it is so enacted.

COMMITTEE REPORT.

COMMITTEE ROOM,
AUSTIN, TEXAS, January 30, 1909.

*Hon. A. M. Kennedy, Speaker of the House of Representatives.*

SIR: Your Committee on Criminal Jurisprudence, to whom was referred House Bill No. 16, a bill to be entitled

An Act to amend Article 639 of Chapter 7, Title XV of the Penal Code of the State of Texas, in reference to the punishment for rape, and providing who shall perform castration, and fixing a fee therefor, and declaring an emergency,

Have had the same under consideration, and I am instructed to report the same back to the House, with the recommendation that it pass.

Mr. Hill appointed to make full report on the same.

BUCHANAN, Acting Chairman.

FULL REPORT.

COMMITTEE ROOM,
AUSTIN, TEXAS, January 29, 1909.

*Hon. A. M. Kennedy, Speaker of the House of Representatives.*

SIR: Having been appointed by your Committee on Criminal Jurisprudence to make full report on House Bill No. 16, a bill to be entitled

An Act to amend Article 639 of Chapter 7, Title XV of the Penal Code of the State of Texas, in reference to the punishment for rape, and providing who shall perform castration, and fixing a fee therefor, and declaring an emergency,

Beg leave to submit the following:

The frequency with which the crime of rape is committed and the apparent inadequacy of present punishment, calls for more drastic measures. It is believed that the provisions of this bill will strike directly at the root of the evil; that when these provisions are enacted into statute they will stand as a monument to the wisdom of this Legislature, and will be beyond the wiles of codifiers and recodifiers. This law will apply to his sensualities rather than to his fears, and it is believed the application of this punishment will render him unfit for future operations in this line.

It is also believed that there is no merit in the constitutional objection urged to this bill, that one member can not be punished for the crime of another. We believe that the close proximity of those instrumental in the commission of this crime is conclusive evidence of a conspiracy between them to commit it.

We, therefore, recommend that the bill do pass.

Very respectfully,

HILL.

Under suspension of the rules, the bill passed—78 to 25. It now goes to the Senate, and it is believed that it will pass that body also.—D.

